# Anesthetic management of a patient with Osler-Weber-Rendu syndrome with multiple pulmonary arteriovenous malformations and pheochromocytoma for femoral artificial bone replacement: a case report

**DOI:** 10.1186/s40981-023-00600-4

**Published:** 2023-02-08

**Authors:** Toshiharu Hiyoshi, Kazuyoshi Shimizu, Satoshi Kimura, Toshiki Naritani, Hiroshi Morimatsu

**Affiliations:** Department of Anesthesiology and Resuscitology, Okayama University Hospital, 2-5-1, Shikata-cho, Kita-ku, Okayama, 700-8558 Japan

**Keywords:** Osler-Weber-Rendu syndrome, Arteriovenous malformations, General anesthesia, Neuraxial anesthesia

## Abstract

**Background:**

Osler-Weber-Rendu syndrome is characterized by mucocutaneous telangiectasia and arteriovenous malformations in organs. Anesthesia for patients with Osler-Weber-Rendu syndrome is challenging due to complications and physiological changes.

**Case presentation:**

The case was a 49-year-old female with Osler-Weber-Rendu syndrome, multiple pulmonary arteriovenous malformations and pheochromocytoma who presented for femoral bone head fracture with metastatic adenocarcinoma. The patient was scheduled to undergo bone tumor resection and artificial bone replacement, being positioned laterally with a planned operation duration of 5 h. Anesthesia was managed with spinal and epidural anesthesia, combined with sedation by sevoflurane using a supraglottic airway (SGA) device under spontaneous breathing. Her intraoperative and postoperative courses were uneventful.

**Conclusion:**

Neuraxial anesthesia combined with general anesthesia using an SGA device to maintain spontaneous ventilation in order to minimize the risk of rupture of pulmonary arteriovenous malformations could be an option.

## Background

Osler-Weber-Rendu syndrome, which is also known as hereditary hemorrhagic telangiectasia, is an autosomal dominant disorder characterized by mucocutaneous telangiectasia and arteriovenous malformations. The frequency is estimated to be 1 in 2000–40,000 [1]. Lesions can occur in the nasopharynx, gastrointestinal tract, lung, central nervous system, liver, kidney, and other organs [2]. Possible hemorrhage due to fragility of abnormal vessels as well as respiratory and circulatory complications due to pulmonary and/or systemic shunts can be fatal and require careful anesthetic planning and management. General anesthesia and neuraxial anesthesia have both advantages and disadvantages for patients with Osler-Weber-Rendu syndrome. Anesthesiologists need to make plans based on the sites and severity of the lesions and other comorbidities as well as surgical factors. Here, we report anesthetic management for a patient with Osler-Weber-Rendu syndrome who underwent bone tumor resection and artificial bone replacement in the lateral position.

## Case presentation

A 49-year-old female with a body mass index of 17 kg/m^2^ presented for pathological fracture of right femoral bone head with metastatic adenocarcinoma and was scheduled to undergo bone tumor resection and artificial bone replacement. She was diagnosed with Osler-Weber-Rendu syndrome at the age of 30 and accompanied with multiple pulmonary arteriovenous malformations (AVMs) and pheochromocytoma. She had a history of brain abscess, takotsubo cardiomyopathy and preoperative coil embolization of the AVMs. She did not have any history of recurrent epistaxis or gastrointestinal bleeding.

**Fig. 1:**
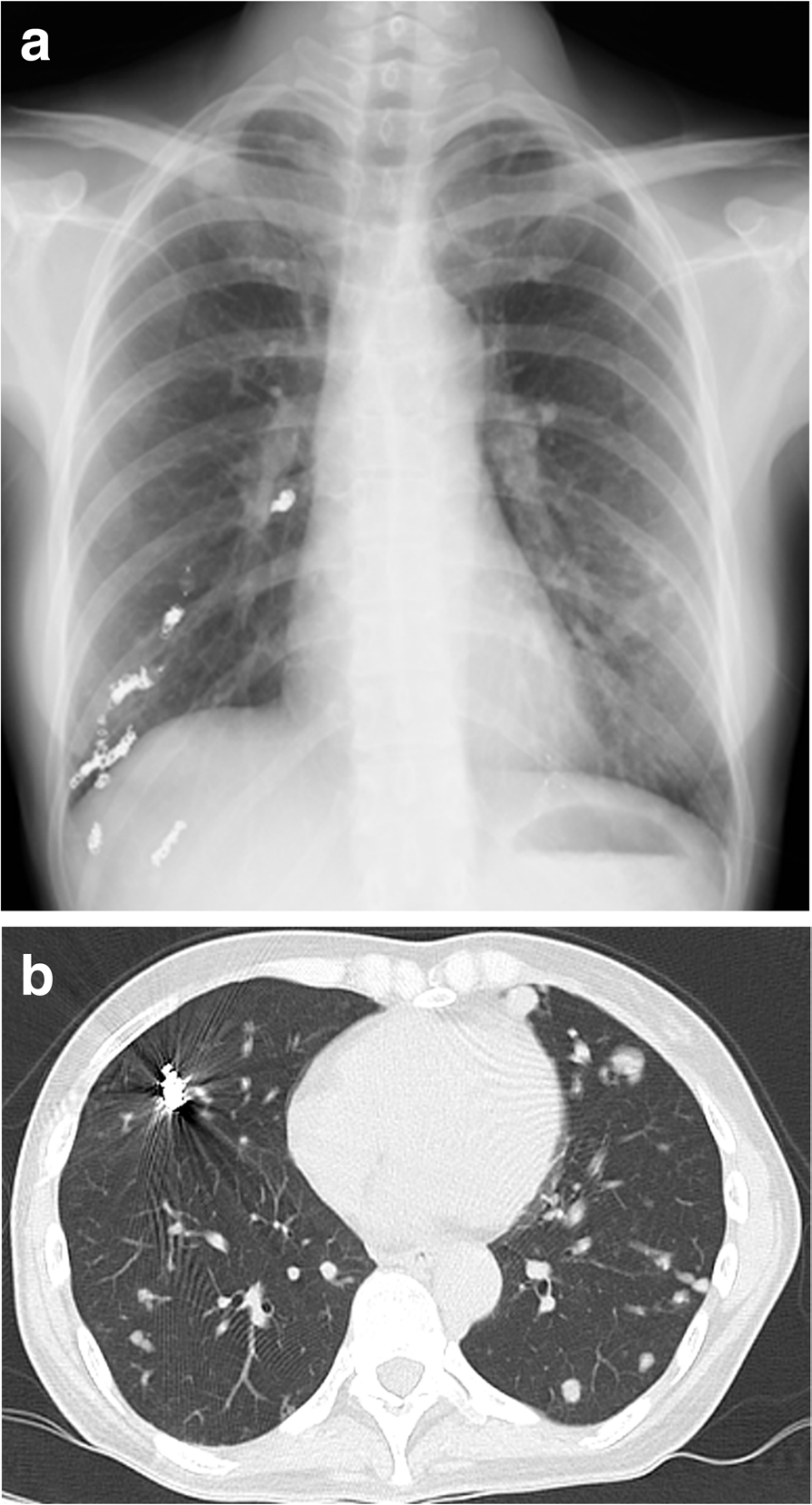
**a** Chest radiograph. The chest radiograph shows preoperative coil embolization of the pulmonary arteriovenous malformations on the right lung. **b** Computed tomography of the chest. The computed tomography of the chest shows bilateral pulmonary arteriovenous malformations

A preoperative physical examination did not reveal any special finding on her body surface. Blood pressure was 105/64 mmHg, with doxazosin at a dose of 3 mg per day, and heart rate was 97 bpm. Peripheral oxygen saturation (SpO_2_) was 86% in room air, possibly due to the remaining pulmonary AVMs, but the Hugh-Jones classification was class I. Blood tests were remarkable only for hemoglobin level of 11.2 g/dL. Urinary adrenaline, noradrenaline and dopamine levels were 14.2 mcg/day, 965.8 mcg/day and 807.3 mcg/day, respectively. An electrocardiogram (ECG) showed negative T-waves on V3-5 and a transthoracic echocardiogram showed a peripheral left ventricle hypertrophy with an ejection fraction of 67%. Preoperative image examination revealed bilateral pulmonary AVMs (Fig. 1a, b), but no AVM in the brain or in the spinal cord, and a pancreas tail tumor, which was considered to be the origin of the metastatic bone tumor.

Preoperative estimation of the operation duration was 5 h, and estimated intraoperative blood loss was 500 mL. She was in anxiety and wished to have sedation during the procedure.

Combined spinal and epidural anesthesia was planned in order to reduce the risk of rupture of the pulmonary AVMs due to inadequate pain control and positive pressure mechanical ventilation. We planned to add sedation by general anesthesia with a supraglottic airway device (SGA) to maintain spontaneous ventilation because of the estimated long duration of surgery, her mental status and large amount of blood loss.

**Fig. 2:**
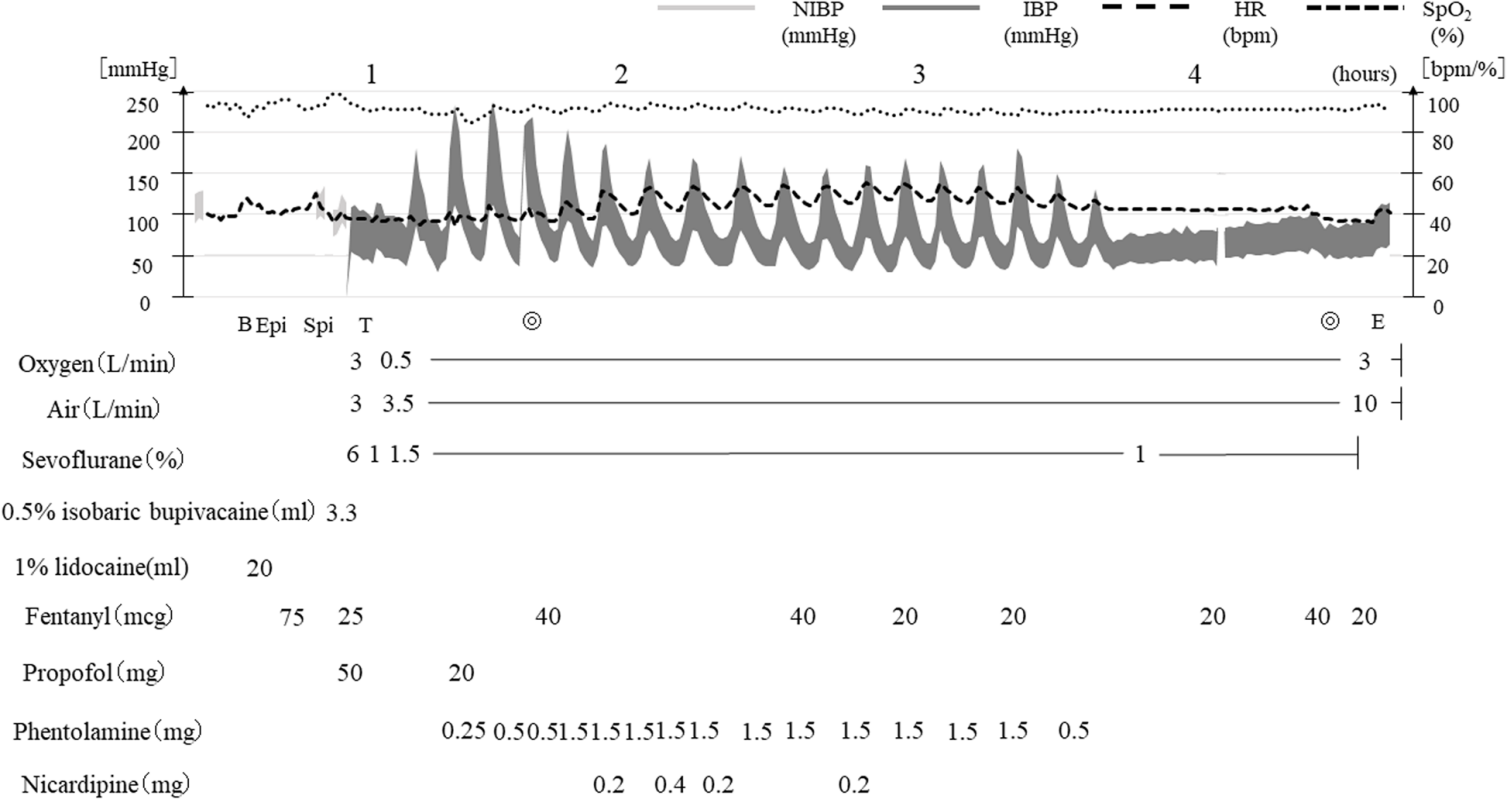
Anesthetic record. Blood pressure fluctuated during the procedure, necessitating frequent boluses of phentolamine and nicardipine. Abbreviations: NIBP, non-invasive blood pressure; IBP, invasive blood pressure; HR, heart rate; SpO_2_, peripheral oxygen saturation; B, pericapsular nerve group block; Epi, epidural catheter insertion; Spi, spinal anesthesia; T, insertion of supraglottic airway device; E; removal of supraglottic airway device; ◎, start and end of operation

After entering the operating room, a peripheral venous line was secured, and a pericapsular nerve group block was performed using 20 mL of 1% lidocaine in order to relieve the pain of transferring between beds in the operating room and of positioning for spinal and epidural anesthesia (Fig. 2). Then the patient was positioned laterally on the left side, an epidural catheter was inserted at the L2-3 lumbar intervertebral space, and spinal anesthesia was performed at the L3-4 lumbar intervertebral space using 3.3 mL of 0.5% isobaric bupivacaine. After changing to the supine position and confirming Th10 or lower levels of the block, SGA device (Air-Q®, Cookgas LLC, USA) was inserted with 50 mg propofol and 75 mcg fentanyl, and spontaneous breathing was maintained with concentrations of 1.0–1.5% sevoflurane. Under sedation, a central venous line and an arterial line were secured, and processed regional cerebral oxygen saturation (rSO_2_) was monitored in addition to standard monitoring including 5-lead ECG, pulse oximetry and capnography. Although no major blood pressure changes were observed during induction of anesthesia, her systolic blood pressure fluctuated in the range of 70 to 220 mmHg during the operation, necessitating frequent boluses of phentolamine and nicardipine. Her SpO_2_ and rSO_2_ were stable in the range of 89–92% and 55–60%, respectively, under a fraction of inspired oxygen of 0.3. Neither positive end-expiratory pressure nor pressure support was applied, and respiratory rate of spontaneous breathing was 20–30 per minute with expired carbon dioxide concentration of 30–38 mmHg during the procedure. There was no difficulty or trouble in managing her airway with an SGA device in the lateral position. No epidural local anesthetic agent was required, and the total amounts of fentanyl used during the procedure was 300 mcg. The surgery was competed in 170 min and the total amount of hemorrhage was 630 mL. A total of 1953 mL of Ringer’s solution and 4 units of red blood cells were administered during the procedure with 630 mL of urine output. After confirming emergence from anesthesia without a new neurological abnormality, the SGA device was removed in the operating room.

After the operation, she was transferred to the intensive care unit (ICU) as planned. Her pain was well controlled with patient-controlled epidural analgesia. Her postoperative course was uneventful, and she was discharged from the ICU on postoperative day 1.

## Discussion

Osler-Weber-Rendu syndrome presents a wide variety of manifestations due to arteriovenous malformation and telangiectasis, most commonly in the nasal mucosa (> 90%), followed by the gastrointestinal tract (50–80%), lung (> = 20%), brain (15%), and liver (8–16%) [2]. Patients with this syndrome have a risk of epistaxis, gastrointestinal hemorrhage, pulmonary hemorrhage, and cerebral hemorrhage [3]. One of the main anesthetic considerations is controlling blood pressure with adequate sedation and analgesia in order to prevent bleeding from AVMs and telangiectases, which was crucially important in our case because of the existence of pheochromocytoma. Since systemic AVMs can have left-to-right shunting and decreased systemic vascular resistance, hypotension secondary to anesthetic agents and/or bleeding could be a problem with an unpredictable response to the vasoactive drugs [3–5]. In addition, low systemic vascular resistance due to AVMs could also result in depressurized circulation and high output heart failure [6, 7]. Pulmonary AVMs lead to low arterial oxygen saturation, the severity of which depends on right-to-left shunt fraction. The right-to-left shunt can also cause paradoxical emboli, leading to systemic organ infarctions even by small air bubbles or thrombosis [8]. Our patient had residual pulmonary AVMs with low arterial oxygen saturation and had a history of brain abscess, possibly due to the right-to-left shunt.

General anesthesia for patients with Osler-Weber-Rendu syndrome has several disadvantages. First, general anesthesia with positive pressure ventilation may cause rupture of pulmonary AVMs, which is a fatal complication of the disease [9, 10]. Positive pressure ventilation also increases pulmonary vascular resistance, causing hypoxia and paradoxical emboli due to the exaggeration of right-to-left shunt. Second, laryngoscopy and intubation maneuvers might cause bleeding as telangiectasias can be found in both the upper and lower airway tract [9]. Unconsciousness under general anesthesia also has a disadvantage in terms of neurological monitoring for patients with a risk of cerebral hemorrhage and paradoxical emboli. Although regional anesthesia solves some of the issues with general anesthesia, it is not risk-free. It has been reported that spinal anesthesia was performed for a case with AVM in the spinal cord and that neurological change caused by the epidural hematoma was found after the operation [11]. Therefore, the presence of spinal AVM is considered to be a relative contraindication to neuraxial anesthesia [12, 13] Furthermore, hypotension secondary to sympathectomy from spinal anesthesia in addition to low systemic vascular resistance from systemic AVMs is another issue. Patients with pheochromocytoma may have more profound hypotension because of a highly contracted intravascular volume secondary to the chronic catecholamine release [14].

For selecting an anesthesia technique, the risks of general anesthesia and regional anesthesia as discussed above should be balanced with consideration of factors from the surgical procedure. We performed spinal and epidural anesthesia in order to avoid positive pressure ventilation and to provide adequate analgesia for a prolonged duration of surgery. Considering the possible long operation duration with the lateral position on the left side and with a risk of bleeding in the surgical field, sedation during the procedure was preferable. We decided to use sevoflurane with an SGA device to ensure spontaneous ventilation. While the use of an SGA device has a risk of upper airway bleeding, it enables possible emergent tracheal intubation through the laryngeal mask, even in the lateral position, in the case of respiratory and/or circulatory collapse during the procedure.

In summary, anesthesia management for Osler-Weber-Rendu syndrome is challenging. Both general anesthesia and neuraxial anesthesia including spinal and/or epidural anesthesia have advantages and disadvantages. For surgery with a long duration in the lateral position, neuraxial anesthesia combined with general anesthesia using an SGA device to maintain spontaneous ventilation could be an option.

## Data Availability

Not applicable.

## Abbreviations

AVM: Arteriovenous malformations
SpO_2_: Peripheral oxygen saturation
ECG: Electrocardiogram
rSO_2_: Regional cerebral oxygen saturation
